# Environmental Risk and Risk of Resistance Selection Due to Antimicrobials’ Occurrence in Two Polish Wastewater Treatment Plants and Receiving Surface Water

**DOI:** 10.3390/molecules25061470

**Published:** 2020-03-24

**Authors:** Joanna Giebułtowicz, Grzegorz Nałęcz-Jawecki, Monika Harnisz, Dawid Kucharski, Ewa Korzeniewska, Grażyna Płaza

**Affiliations:** 1 Department of Bioanalysis and Drugs Analysis, Faculty of Pharmacy, Medical University of Warsaw, 1 Banacha, 02-097 Warszawa, Poland; jgiebultowicz@wum.edu.pl (J.G.); dawid.kucharski@wum.edu.pl (D.K.); 2 Department of Environmental Health Sciences, Faculty of Pharmacy, Medical University of Warsaw, 1 Banacha, 02-097 Warszawa, Poland; grzegorz.nalecz-jawecki@wum.edu.pl; 3 Department of Environmental Microbiology, Faculty of Environmental Sciences, University of Warmia and Mazury, 5 Oczapowskiego, 10-719 Olsztyn, Poland; monikah@uwm.edu.pl (M.H.); ewa.korzeniewska@uwm.edu.pl (E.K.); 4 Microbiology Unit, Institute for Ecology of Industrial Areas, 6 Kossutha, 40-844 Katowice, Poland

**Keywords:** antibiotics, wastewater, sewage sludge, risk assessment, removal efficiency, LC-MS/MS analysis

## Abstract

In this study, a screening of 26 selected antimicrobials using liquid chromatography coupled to a tandem mass spectrometry method in two Polish wastewater treatment plants and their receiving surface waters was provided. The highest average concentrations of metronidazole (7400 ng/L), ciprofloxacin (4300 ng/L), vancomycin (3200 ng/L), and sulfamethoxazole (3000 ng/L) were observed in influent of WWTP2. Ciprofloxacin and sulfamethoxazole were the most dominant antimicrobials in influent and effluent of both WWTPs. In the sludge samples the highest mean concentrations were found for ciprofloxacin (up to 28 μg/g) and norfloxacin (up to 5.3 μg/g). The removal efficiency of tested antimicrobials was found to be more than 50% for both WWTPs. However, the presence of antimicrobials influenced their concentrations in the receiving waters. The highest antimicrobial resistance risk was estimated in influent of WWTPs for azithromycin, ciprofloxacin, clarithromycin, metronidazole, and trimethoprim and in the sludge samples for the following antimicrobials: azithromycin, ciprofloxacin, clarithromycin, norfloxacin, trimethoprim, ofloxacin, and tetracycline. The high environmental risk for exposure to azithromycin, clarithromycin, and sulfamethoxazole to both cyanobacteria and eukaryotic species in effluents and/or receiving water was noted. Following the obtained results, we suggest extending the watch list of the Water Framework Directive for Union-wide monitoring with sulfamethoxazole.

## 1. Introduction

The fate of contaminants, particularly pharmaceutically active compounds (PhACs) in the environment is receiving considerable attention from researchers. PhACs appear as contaminants in wastewater, soil, surface and ground water, municipal sewage, and in the influents and effluents of wastewater treatment plants [1,2,3]. There are several sources of PhACs in the environment. The most important is human and veterinary medicine as well as plant agriculture. The main sources of aquatic contamination with human antimicrobials are wastewater treatment plants (WWTPs). The PhACs enter WWTPs along with wastewater from the disposal of unused or expired drugs in toilets. However, human excretion is considered to be the most important source. Generally, WWTPs are not designed to eliminate PhACs during the technological process, and a number of studies have shown the presence of different PhACs in both raw and treated sewage sludge and wastewater [4,5,6,7,8]. There is no data on either the removal efficiency or the concentration of antimicrobials in Polish WWTPs. The concentration of PhACs in the environment depends on the consumption of pharmaceuticals, which is country- and culture-specific, and their pharmacokinetics, and may considerably vary with seasons and physicochemical properties of these compounds, various process operating parameters of WWTPs, and bacterial community structure [9,10]. According to the European Centre for Disease Prevention, in 2018 the corresponding population-weighted mean consumption of antimicrobials (in defined daily dose (DDD) units per 1000 inhabitants per day) in European Union and European Economic Area countries was 18.4 DDD. In Poland the consumption rate was calculated as 23 DDD. Higher values were observed only for France (23.6 DDD), Greece (32.4 DDD), Romania (25.0 DDD) and Spain (24.3 DDD) [11].

Antimicrobials are one of the most extensively investigated PhACs. They belong to contaminants of emerging concern (CEC), which helps assess hazards to human health and ecosystems. They are one of the most popular pharmaceuticals used in veterinary care, farming, and medicine. According to the United States Geological Survey (USGS), CEC includes “any synthetic or naturally occurring chemical or any microorganism that is not commonly monitored in the environment, but has the potential to enter the environment and cause known or suspected adverse ecological and/or human health effects” [12].

WWTPs are not specifically designed for antimicrobial removal, and, consequently, these molecules are released directly into the receiving environment. An important issue is to identify the sources of antimicrobials in water and to assess their concentrations in surface, ground, and potable waters. The presence of antimicrobials in surface and ground waters, and even in drinking water, has been identified worldwide, for example in the UK [13], Italy [14], China [15], Australia [16], and the USA [17]. In our previous study, 20 of the 26 investigated antimicrobials up to a concentration of 1000 ng/L in the river water close to the effluent discharge from the main WWTP in Warsaw (Poland) were detected [18]. Although WWTPs are considered the main source of antimicrobials for surface waters, the current legislation at a European level does not contain an antimicrobial concentration requirement for discharge from WWTPs to receiving water. Antimicrobials have been determined in numerous WWTPs such as those in Germany [8,19], France [7], Croatia [20], Spain [21], China [22], Switzerland [23], Sweden [24], and Norway [25]. Given the number of scientific papers regarding the analysis of the antimicrobials’ concentrations in European and global WWTPs, the knowledge of the occurrence of antimicrobials in Polish WWTPs is scare. Moreover, there are only scarce data on risk assessment on resistance selection and on environmental toxicity in WWTPs. To our best knowledge, no such data regarding sludge and sludge-affected soils exists.

The presence of the antimicrobials in the environment may pose an environmental risk. Environmental risk is defined as actual or potential threat of adverse effects on aquatic and/or terrestrial organisms. In the case of antimicrobials, the most endangered are prokaryotes, e.g., nitrification bacteria [26] or cyanobacteria [27]. Antimicrobials can also pose a risk of resistance selection. It is observed as preferential outgrowth of antimicrobial-resistant bacteria and changes in the antibiotic sensitivity of the entire microbial population in antimicrobial concentrations below the minimal inhibitory concentration. As a consequence, the resistant bacteria is able to survive in the presence of an antimicrobial in concentration that is usually sufficient to inhibit or kill microorganisms of the same species [28]. The antimicrobial-resistant genes can be transferred between distantly related bacterial species and to bacteria that colonize the human body and human pathogens [26]. The estimates suggest that 700,000 deaths occur every year because of antimicrobial resistance; moreover, by 2050, there might be 10 million deaths every year [29].

In this context, the aim of the study was to investigate the occurrence, abundance, and removal efficiency of the selected antimicrobials in two Polish wastewater treatment plants. The risk assessment approach based on environmental risk quotients (RQs) was also calculated to assess antimicrobial resistance risks and ecological environmental risk of antimicrobials to cyanobacteria and eukaryotic species. The antimicrobials were selected based on the sales data and occurrence of the antimicrobials in the environment in Europe. Additionally, the satisfactory performance of the analytical method was taken into account. 

## 2. Results

### 2.1. Predicted Concentrations of Antimicrobials in WWTPs

According to sales data for 2018 (published by the Polish National Health Fund (NFZ)), among detected antimicrobials, the highest sale in Poland was noted for clarithromycin (up to 1509 kg/month), sulfamethoxazole (up to 1358 kg/month), ofloxacin (up to 829 kg/month), ciprofloxacin (up to 821 kg/month), and clindamycin (737 kg/month). We compared the predicted load of antimicrobials in the WWTP1 (PLoad) (Poland) with the load calculated based on the measured concentrations of the drugs in the influent (water phase) and in the primary sludge (Load_W+S_) (Table A1, Online Resource). For most of the tested antimicrobials, the Load_W+S_ to PLoad ratio was low (up to 20%) because the high metabolism in the human body results in a lower level of parent compound, e.g., the biotransformation ratio of clindamycin is 85% [30]. Moreover, the measured load of fluoroquinolones (ciprofloxacin and norfloxacin) was very close to that of the predicted load, and unlike other antimicrobials that are primarily present in the water phase, ciprofloxacin and norfloxacin are distributed evenly between water and the primary sludge. For six antimicrobials (erythromycin, metronidazole, oxytetracycline, tetracycline, sulfathiazole, and vancomycin), the Load_W+S_ to PLoad ratio was very high and exceeded 1000% because of the low and very low predicted load value. Note that almost all antimicrobials in Poland are available by prescription and most of them are reimbursed; however, some, such as vancomycin, are primarily used in hospitals, while tetracyclines (oxytetracycline and tetracycline) and sulfathiazole are primarily used in veterinary medicine and are not reported by NFZ. 

### 2.2. Antimicrobial Concentrations in Influents of WWTPs 

The concentrations of 26 antimicrobials in influents from WWTP1 and WWTP2 and receiving waterbodies are shown in Table 1. The significant differences between the concentrations of antimicrobials in the samples collected in different sampling periods were observed. The differences were due to seasonal variations in antimicrobial use. According to National Health Fund (NFZ) database, in summer the consumption of antimicrobials was low (Table A1). In autumn and winter, the consumption increased significantly, probably due to numerous infections occurring each year in that period [31]. 

Nineteen and 18 out of 26 analyzed antimicrobials were detected in the wastewater from WWTP1 and WWTP2, respectively. In most cases, the antimicrobial levels were higher in wastewater of WWTP2 than in wastewater of WWTP1. The mean values of antimicrobials’ concentration in analyzed samples ranged from <MDL (method detection limit) to 7400 ng/L. In influent from WWTP1, the average concentrations of two antimicrobials, i.e., ciprofloxacin and sulfamethoxazole, were higher than 1000 ng/L and concentrations of 10 antimicrobials was higher than 100 ng/L. While in influent from WWTP2, the concentration of four antimicrobials, i.e., ciprofloxacin, metronidazole, sulfamethoxazole, and vancomycin, exceeded the level of 1000 ng/L and concentrations of 12 antimicrobials exceeded the level of 100 ng/L. The highest average concentrations of antimicrobials in influent from WWTP2 were recorded for metronidazole (7400 ng/L), followed by ciprofloxacin (4300 ng/L), vancomycin (u 3200 ng/L), and sulfamethoxazole (3000 ng/L). In other countries, the concentration of metronidazole was lower than 1000 ng/L, that of ciprofloxacin was up to 3800 ng/L [32] but frequently below 400 ng/L [33], and that of sulfamethoxazole was up to 7900 ng/L [34]. There are not much data on vancomycin occurrence in the environment, and it has not been detected in very high concentrations to date as it is primarily used intravenously to treat severe infections in hospitals [35]. The differences in the concentrations of antimicrobials in the influent between two WWTPs can be due to various consumption of antimicrobials in the sampling period and various sources of antimicrobials in these regions. As an example, near WWTP1 is a hospital (449 beds), whereas near WWTP2 is a hospital (458 beds), poultry plant, and galenic laboratory.

### 2.3. Antimicrobial Concentrations in Effluent of WWTPs

**Figure 1:**
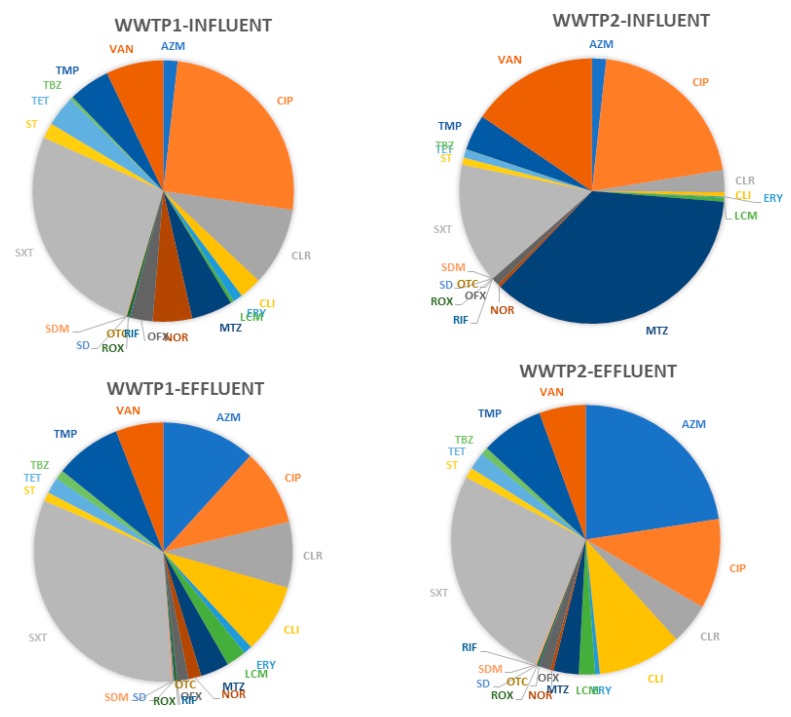
Percentage contribution (%) of tested antimicrobials in influent and effluent of both WWTPs, *n* = 3.

The concentrations of 26 antimicrobials in effluents from WWTP1 and WWTP2 are shown in Table 1. In effluents from both WWTPs, the highest average concentrations were observed for azithromycin (up to 650 ng/L), sulfamethoxazole (up to 770 ng/L), ciprofloxacin (up to 312 ng/L), and clindamycin (up to 290 ng/L). In effluent from WWTP2, a higher mean concentration of azithromycin but lower of metronidazole was observed. The percentage contribution of the analyzed antimicrobials in influent and effluent of both WWTPs are presented in Figure 1. To summarize, among antimicrobials tested, ciprofloxacin and sulfamethoxazole were the most dominant antimicrobials in influent and effluent from both WWTPs. 

Comparing our data from the literature, the results obtained were similar. The concentration of azithromycin was up to 380 ng/L [36] in Switzerland, that of sulfamethoxazole was up to 1300 ng/L in Spain [21], and that of clindamycin was up to 5 ng/L in Australia [32].

### 2.4. Antimicrobial Concentrations in Receiving Waters 

In order to assess the impact of antimicrobials on the receiving water bodies, samples were taken upstream and downstream from the WWTPs’ discharge (see Section 3.1). The concentrations of 26 antimicrobials in receiving water are shown in Table 1. The river upstream of WWTP1 was more polluted with analyzed antimicrobials than WWTP2 upstream river (Table 1). The highest concentration of sulfamethoxazole (average value: 644 ng/L) was detected upstream of WWTP1. While in the upstream of WWTP2, the highest concentration of clarithromycin (average value: 12.2 ng/L) was detected. The wastewater discharge from WWTP2 resulted in a significant increase of antimicrobials’ concentration in the river. This fact can be explained by the high concentrations of antimicrobials in the case of effluent from WWTP2 (Table 1). In the case of WWTP1, taking into account all antimicrobials, no statistical differences between upstream and downstream were observed. Despite this, the average concentration of azithromycin in WWTP1 downstream was more than 15 times higher compared to the concentration in the upstream. The concentrations of antimicrobials in the receiving waters depend on the concentration of antimicrobials in effluent, distance of sampling, or the different flow rate. In our study, WWTP2 effluent effect on antimicrobials’ concentrations in the receiving surface water was observed.

### 2.5. Antimicrobials’ Removal Efficiency

**Figure 2:**
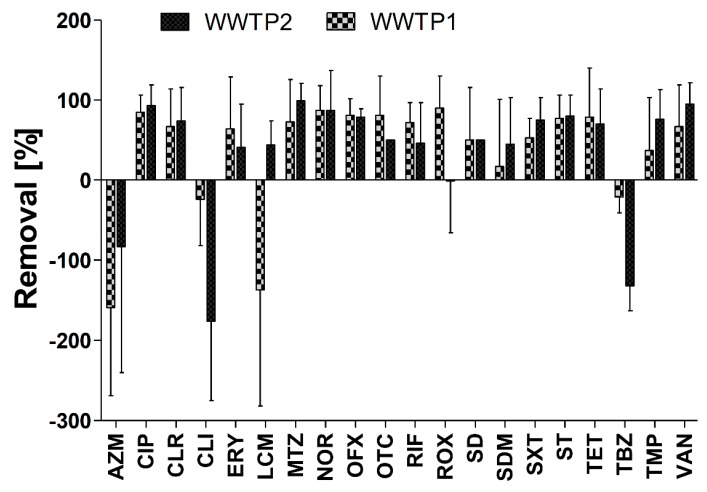
Removal efficiency (%) of selected antimicrobials in WWTPs calculated based on equations described in Section 3.3.

The removal efficiency (calculated based on equations described in Section 3.3) of tested antimicrobials was similar for both WWTPs (*p* > 0.05). The average removal efficiency was above 50% for 12 and 10 out of 20 detected antimicrobials for WWTP1 and WWTP2, respectively (Figure 2). Among the antimicrobials from the sulfonamide group, four were detected in effluents and their removal rate ranged from 17% to 80% with an average of 52%. Certain inconsistencies exist in literature about sulfonamide removal, as per the review by Le-Minh et al. [37]. Some researchers have reported an effective removal of sulfonamide [38], although others have mentioned the opposite results [39]. Based on the literature data, this fact might be explained by the differences in operational conditions of each WWTP. 

The removal efficiency of trimethoprim was 37% in WWTP1 and 76% in WWTP2 (*p* = 0.2) (the treatment process used in the WWTPs are presented in Section 3.1). Regarding fluoroquinolones, three of them, i.e., norfloxacin, ciprofloxacin, and ofloxacin, were detected in influent of both WWTPs with the average removal being above 80%. The plausible mechanism for the removal of fluoroquinolones from water is sorption to sediment because their concentration was detected as very high in the sludge [40,41,42] and a slow biodegradation rate was reported [43]. The average removal efficiency for fluoroquinolone antimicrobials estimated by Wang et al. [44] was about 50%. Rodayan et al. [45] reported values of around 60%, while for WWTPs in Switzerland, higher removal efficiencies were observed, reaching up to 87% (for norfloxacin) [46]. In the study of Gao et al. [47], the removal efficiencies of WWTPs for fluoroquinolone antimicrobials ranged from 48% to 72%.

In this study, a negative removal rate for azithromycin, clindamycin, lincomycin, and thiabendazole was observed (Figure 2). Moreover, for these antimicrobials, a high variance in removal rate was noted, which agrees with previous studies in which certain antimicrobials were reported to be more abundant in effluents than influents [6,8,10,25,33,48].

The negative removal rate has been detected in the literature. For example, Chunhui et al. [49] reported that the removal efficiencies for macrolide antimicrobials in WWTPs was –4%. Similarly, Gao et al. [47] reported that the removal rate of macrolide antimicrobials was higher, and reached from –34% to 69%. The negative removal rate is explained in many ways. First, certain metabolites can return to the parent pharmaceutical during primary and secondary treatment because of glucuronide conjugate hydrolytic cleavage. Second, pharmaceuticals that sorb to organic matter and particles, as well as accumulate in sediments and biofilms, might be resuspended in wastewater during storm water events. Typically, deconjugation or resorption from particles is observed for fluoroquinolones (ciprofloxacin, ofloxacin, and norfloxacin), lincomycin, tetracycline, trimethoprim, and sulfamethoxazole. This issue is particularly relevant to pharmaceuticals that are mainly excreted with bile and feces-like macrolides. During wastewater treatment, they are redissolved, and, therefore, their concentrations in water increase [10,33].

**Figure 3:**
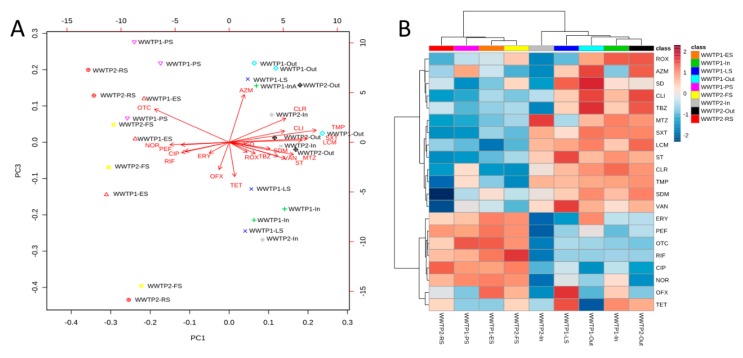
Differences in antimicrobials’ concentrations between the wastewater, sludge samples, and leachate collected from WWTP1 and WWTP2. Principal component analysis (PCA) of the all samples (**A**). Variables of a data matrix are represented as arrows. Visualization of the observed relations on the heat map (**B**). ES, excessive sludge; FS, fermented sludge; In, influent; LS, leachate; Out, effluent; PS, primary sludge; RS, residual sludge.

HRT (hydraulic retention time) is one of the major factors influencing the antimicrobial removal efficiency, particularly for compounds that are readily biodegradable and have a low K_d_ (low tendency to absorb to sludge) [50]. In this study, HRT was 12 h in WWTP1 and 24 h in WWTP2. Other factors influencing the removal efficiency are adsorption coefficients and persistence of the antimicrobials. Lower levels in effluents could be also interpreted as the removal of antimicrobials because of biodegradation and/or chemical and physical transformations, e.g., hydrolysis or sorption to solid matter. Biodegradation/biotransformation and sorption are the two primary mechanisms occurring in the WWTPs. The sorption to sewage sludge is the primary removal mechanism for certain antimicrobials in the samples, e.g., the percentage of the daily load of antimicrobials was >40% for azithromycin, ciprofloxacin, ofloxacin, and oxytetracycline in sludge (Table A2), while the lowest value was observed for sulfamethoxazole and lincomycin. The differences between the sorption of antimicrobials depend on their physicochemical properties, such as charge and lipophilicity, the properties of the sludge-like chemical nature, and other factors such as pH and redox potential [51]. Sorption may occur by hydrophobic interactions with lipophilic cell membranes of the microorganisms or the lipid fractions of the suspended solids and electrostatic interactions of positively charged groups of chemicals with negatively charged surfaces of microorganisms or other components of the sludge [33]. Figure 3 shows differences between the concentrations of antimicrobials in wastewater and sludge (separation observed by principal component 1 (PC1) accounted for 45% of total variance). Two groups were designated by the various amounts of oxytetracycline and norfloxacin in the samples. The relative percentage estimated for the antimicrobial was significantly higher in the sludge than in the wastewater. In wastewater, the relative percentage of metronidazole, lincomycin, and sulfamethoxazole was higher compared to that in the sludge. Similarly, a higher contribution of metronidazole and erythromycin in wastewater and ciprofloxacin in sludge was observed by Ostman et al. [24].

Our results showed that leachate from WWTP1 contained a significant amount of antimicrobials. The mean concentrations of nine antimicrobials were above 100 ng/L (Table 1). The concentrations of azithromycin, ciprofloxacin, norfloxacin, ofloxacin, sulfamethoxazole, and vancomycin were comparable to those in the WWTP1 influent. The results obtained suggest that the antimicrobials are strongly sorbed to solids (Table A1). The antimicrobials bound to solids can be leached to the environment, which should be taken into consideration when conducting the risk assessment. In the Table A1, the K_d_ values for tested antimicrobials are presented. Generally, K_d_ is used to estimate the mobility and distribution of the pharmaceuticals in the environment [41,52]. Compounds with low K_d_ are potentially mobile from soil to the water (through leaching or runoff). According to our study, the most mobile antimicrobials are trimethoprim (log K_d_ = 5.8), sulfamethoxazole (log K_d_ = 5.1), lincomycin (log K_d_ = 5.2), clarithromycin (log K_d_ = 5.7), clindamycin (log K_d_ = 5.7), and thiabendazole (log K_d_ = 5.9).

### 2.6. Antimicrobials’ Concentrations in Sewage Sludge from WWTPs

Fifteen antimicrobials in the primary (WWTP1-PS) and excessive sludge (WWTP1-ES) from WWTP1 as well as in the fermented (WWTP2-FS) and residue sludge (WWTP2-RS) from WWTP2 were detected (Table 2). Results obtained indicated that a part of the antimicrobials were adsorbed to the sludge along the whole technological process.

In our study the highest concentrations for fluoroquinolones, such as ciprofloxacin (up to 57 µg/g), norfloxacin (up to 9.5 µg/g), ofloxacin (up to 2.0 µg/g), and azithromycin (up to 2.3 µg/g), were determined in the sludge samples (data not presented). In China, the concentration of norfloxacin, ofloxacin, and ciprofloxacin in sludge was determined from all the major provincial cities, ranging ranged from 0.1 to 15.7 µg/g, from 0.3 to 7.9 µg/g, and from 0.1 to 4.7 µg/g, respectively [53]. In Switzerland (up to 3.3 µg/g) and Sweden (up to 4.2 µg/g), the maximum concentration of norfloxacin was lower than that in China and presented in Poland. Furthermore, the maximum level of other fluoroquinolones was higher in Poland than in Switzerland (up to 0.9 µg/g for ciprofloxacin) and Sweden (up to 4.8 µg/g for ciprofloxacin and up to 2.0 µg/g for ofloxacin) [54,55]. The concentration of azithromycin in the activated sludge was up to 0.16 µg/g and 0.056 µg/g in Germany and Switzerland, respectively, and the values were lower compared to those for Poland (up to 0.71 µg/g) [56]. Recently, a study from Germany reported the concentration of azithromycin and ciprofloxacin in sediments to be up to 0.33 µg/g and 0.71 µg/g [41], respectively. 

### 2.7. Antimicrobial Resistance Risk Assessment

**Figure 4:**
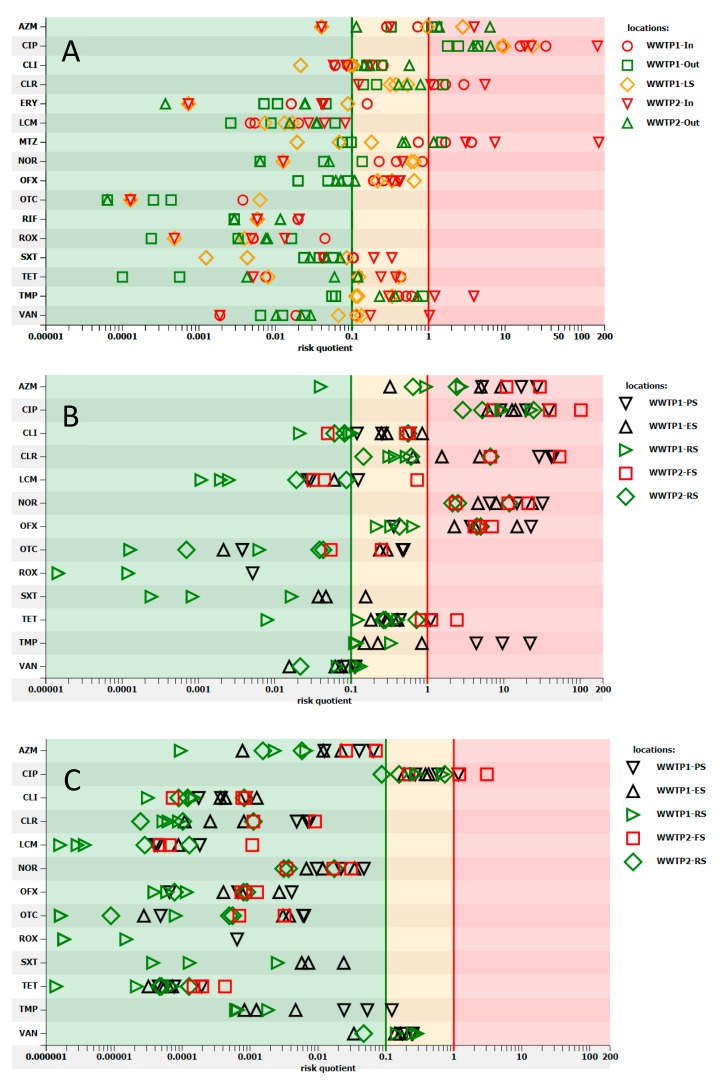
Estimation of risk quotients of resistance selection for the antimicrobials occurring in wastewater (**A**), sludge (**B**), and sludge-amended soil (**C**). Risk quotient below 0.1 indicates minimal risk (green area), between 0.1 and 1 is medium risk (orange area), and over 1 is high risk (red area). ES, excessive sludge; FS, fermented sludge; In, influent; LS, leachate; Out, effluent; PS, primary sludge; RS, residual sludge.

The risk factor of antimicrobial resistance selection (named as antimicrobial resistance risk, defined as preferential outgrowth of antimicrobial-resistant bacteria) in wastewater was high for the following antimicrobials: azithromycin, ciprofloxacin, clarithromycin, metronidazole, and trimethoprim (Figure 4A). As expected, this factor was the highest in influent from both WWTPs. Similar results were obtained by Ostman et al. [24], who observed a high risk of resistance selection in influents in Swedish WWTPs because of the prevalence of ciprofloxacin and metronidazole. No risk for azithromycin was detected in their study.

It is interesting, the notable antimicrobial resistance risk was also predicted upstream of both WWTPs. High antimicrobial resistance risk for ciprofloxacin and medium risk for azithromycin, clarithromycin, and clindamycin was observed in the case of WWTP1, while medium antimicrobial resistance risk for ciprofloxacin was noted in the case of WWTP2. Downstream WWTP1, on top of the previous risk, the risk for metronidazole turned medium, whereas the risk for azithromycin rose to high. In the case of WWTP2, the risk for ciprofloxacin rose from medium to high and the medium risk for ofloxacin, clarithromycin, and clindamycin appeared (data not presented).

The antimicrobial resistance risk was high for azithromycin, ciprofloxacin, clarithromycin, norfloxacin, trimethoprim, ofloxacin, and tetracycline in sludge (Figure 4B). Interestingly, residual sludge could pose a risk to the environment, particularly because of the presence of fluoroquinolones and macrolides. Mean PEC_soil_ (predicted environmental concentration in soil) was mainly lower than 1 ng/g and higher concentrations were observed for ciprofloxacin (up to 41 ng/g), norfloxacin (up to 7.8 ng/g), ofloxacin (up to 1.2 ng/g), and azithromycin (up to 2.3 ng/g) (Table A3). When RQ was analyzed, a high risk for soil was noted for ciprofloxacin (Figure 4C). The data on risk assessment on the antimicrobial resistance selection in sludge and sludge-amended soil presented in this paper are the first.

### 2.8. Environmental Risk Assessment 

**Figure 5:**
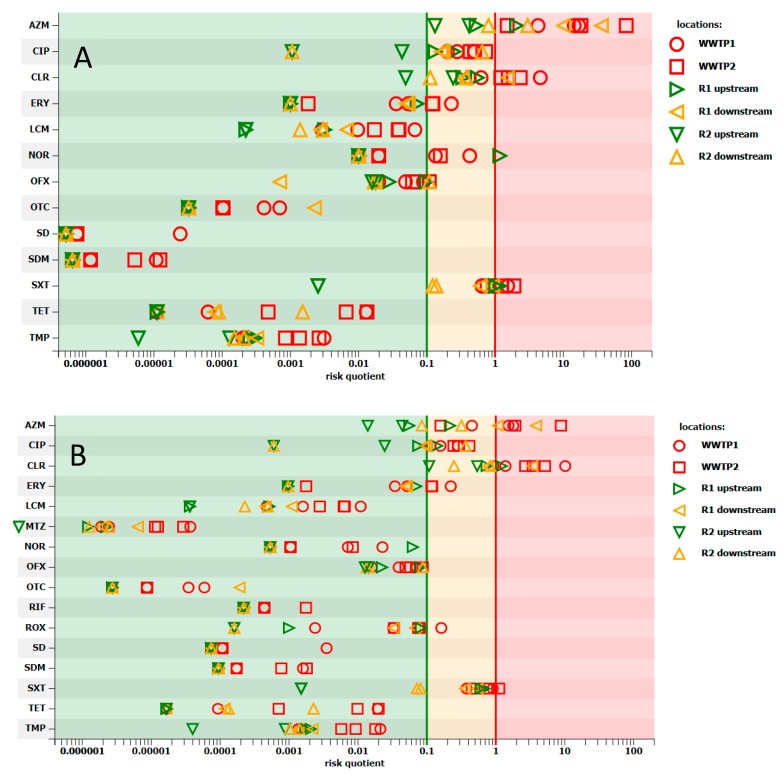
Estimation of risk quotients of chronic exposure of cyanobacteria (**A**) and eukaryotic organisms (**B**) to the antimicrobials occurring in effluents and receiving water. Calculations were based on the measured concentration of antimicrobials in wastewater and PNEC calculated based on [27]. Risk quotient below 0.1 indicates minimal risk (green area), between 0.1 and 1 is medium risk (orange area), and over 1 is high risk (red area).

The high risk was observed for both cyanobacteria (Figure 5A) and eukaryotic species (Figure 5B) due to azithromycin, clarithromycin and sulfamethoxazole in effluents. Furthermore, medium risk was predicted for chronic exposure of cyanobacteria to ciprofloxacin, erythromycin, norfloxacin, and ofloxacin (in effluents). The same level of risk was evaluated for chronic exposure of eukaryotic organisms to ciprofloxacin, erythromycin, and roxithromycin (in effluents). Verlicchi et al. [33] published a review, which reported that six antimicrobials posed a high environmental risk for eukaryotes, i.e., erythromycin, ofloxacin, sulfamethoxazole, clarithromycin, tetracycline, and azithromycin. Harrabi et al. [6] observed a high risk for ofloxacin, ciprofloxacin, azithromycin, sulfamethoxazole, and trimethoprim for eukaryotic species; however, clarithromycin was not evaluated. In their studies, the predicted no effect concentration (PNEC) was, however, calculated differently compared to our study. The authors obtained PNEC values 1000 times lower than the toxicity values found for the most sensitive eukaryotic species that were assayed [6,33]. In our study, other factors, i.e.1000, 100, 50 or10, were used to calculate PNEC. Thus, RQ values obtained for the same concentrations of antimicrobials were lower.

In the case of upstream of WWTP1, there was high risk for cyanobacteria posed by azithromycin, norfloxacin, and sulfamethoxazole, whereas medium risk was observed for ciprofloxacin and clarithromycin. The risk for eukaryote was also observed but the level of the risk was different for the antimicrobials, e.g., for clarithromycin high risk was estimated, and for azithromycin, ciprofloxacin, and sulfamethoxazole medium risk was noted.

None of the antimicrobials posed high risk from upstream of WWTP2. However, medium risk was posed by azithromycin, clarithromycin for cyanobacteria, and medium risk for eukaryote posed by clarithromycin were observed. The medium risk for cyanobacteria posed by ciprofloxacin, ofloxacin, clarithromycin, and sulfamethoxazole in WWTP2 downstream was noted. The risk of toxicity due to azithromycin content increased from medium to high comparing the upstream and downstream. Two more antimicrobials, i.e., azithromycin and ciprofloxacin, were predicted to cause the medium risk for eukaryote.

The RQ calculated for cyanobacteria exposed to sludge was medium for azithromycin (primary sludge up to 0.36, excessive sludge up to 0.12, fermented sludge up to 0.39) and clarithromycin (primary sludge up to 0.13, fermented sludge up to 0.16). No data on risk assessment on antimicrobial presence in sludge was found in literature to compare with our data. 

Antimicrobials are designed to act on prokaryotic organisms; thus, environmental bacteria are more likely to be adversely affected compared to other environmental species such as aquatic invertebrates and vertebrates. However, compared to cyanobacteria, certain microalgae and macrophytes are more sensitive to certain antifolate and quinolone antimicrobials [27]. 

## 3. Materials and Methods 

### 3.1. Description of WWTPs and Sample Collection

The raw influent, final effluent, leachate, and sludge samples (i.e., primary sludge and excessive sludge (WWTP1) or fermented sludge and residual sludge (WWTP2)) were collected from two Polish WWTPs during the three sampling campaigns: (1) In June/July 2018, (2) in October 2018, and (3) in December 2018. Simultaneously, the receiving river water samples from upstream and downstream (WWTP1, 0.25 km; WWTP2, 1.8 km) of the outlet were collected. Each sample was collected in triplicate. The rivers’ average annual flow is 3.5 m^3^/s (WWTP1) and 3.7 m^3^/s (WWTP2). 

WWTP1 is located in one of the cities from Metropolitan Association of Upper Silesia, one of the largest urban areas in the EU and the center of Poland’s industries, particularly coal and metal, with a density of 1600 people per km^2^ [57], geographical coordinates: N 50° 5ʹ 35.881, E 19° 3ʹ 32.202. WWTP2 is located in the forested and agriculture area of Warmian-Masurian Voivodship in the city with a density of 1960 people per km^2^ [57], geographical coordinates: N 53° 48ʹ 46.700, E 20° 26ʹ 55.800.

In 2018, WWTP1 had an equivalent population of 189,332 inhabitants, the average flow rate was 26,830 m^3^/d, and the plant was operated with a hydraulic retention time (HRT) of ~12 h and a solid retention time (SRT) of 25 days, while WWTP2 served a population equivalent of 250,000 inhabitants, and had a flow rate of 32,130 m^3^/d, HRT of 24 h, and SRT of 87 days. Both WWTPs receive domestic, hospital, and industrial wastewaters and treat wastewater with a mechanical-biological system with elevated removal of nutrients. The biological section of WWTP1 included sequential reactors activated sludge chambers (the system of chambers of different oxygen conditions: anaerobic, anoxic, and aerobic), secondary settling tank, and anoxic chamber. In the case of WWTP2, the biological part included a pre-denitrification chamber, phosphorus removal tank, nitrification/denitrification chambers, and secondary settling tanks. Sewage sludge produced in the WWTP1 was subjected to a thickening process, methane fermentation, and dehydration. The leachate produced during sludge fermentation (WWTP1-FS) was returned to the pumping station and again treated. The sewage sludge from WWTP2 was used as a soil improver. The detailed description and technical parameters of both WWTPs are presented by Buta et al. [58].

All samples were collected in triplicate and placed in the sterile glass bottles in volumes of 1–2 liters. Sludge samples were collected after mechanical concentration (primary sludge, WWTP1-PS), after gravity concentration (excessive sludge, WWTP1-ES), after an open fermentation pool (fermented sludge, WWTP2-FS), and after all processes (residue sludge, WWTP2-RS), i.e., sludge for management. Then the samples were transported to the laboratory on the same day and stored at a temperature of 4 °C until analysis.

### 3.2. Analysis of Antimicrobials’ Concentrations

#### 3.2.1. Chemicals

This study targeted 26 antimicrobials including: Azithromycin (AZM), cefadroxil (CFR), ciprofloxacin (CIP), clarithromycin (CLR), clindamycin (CLI), erythromycin (ERY), fleroxacin (FLRX), lincomycin (LCM), lomefloxacin (LOM), metronidazole (MTZ), nalidixic acid (NAL), norfloxacin (NOR), ofloxacin (OFX), oxytetracycline (OTC), pefloxacin (PEF), rifampicin (RIF), roxithromycin (ROX), sulfadiazine (SD), sulfadimethoxine (SDM), sulfamethazine (SDD), sulfamethoxazole (SXT), sulfathiazole (ST), tetracycline (TET), thiabendazole (TBZ), trimethoprim (TMP), and vancomycin (VAN). All pharmaceutical standards for target antimicrobials were of high purity grade (>90%). All compounds were purchased from Sigma–Aldrich (Darmstadt, Germany). Only trimethoprim was sourced from The National Drug Research Institute in Warsaw, Poland. Isotopically labeled compounds used as mixture of internal standards (1 µg/mL in methanol), i.e., azithromycin-C13, ciprofloxacin-D8, sulfamethoxazole-D4, clindamycin-D4, erythromycin-C13D3, ofloxacin-D8, tetracycline-D6, and trimethoprim-D9 (Toronto Research Chemicals, Toronto, Canada), were added to each sample before extraction (500 µL). Solvents, such as HPLC-grade methanol, acetonitrile (LiChrosolv), and formic acid (98%), were obtained from Merck (Darmstadt, Germany). Moreover, ultrapure water was obtained from a Millipore water purification system (Milli-Q water). All working solutions were prepared prior to analysis.

#### 3.2.2. Preparation of Water and Sewage Sludge Samples

Aqueous samples were filtered through glass fiber filters (GF/C, Whatman, Pittsburgh, PA, USA) and membrane filters (0.2 μm, Sartorius Goettingen, Germany). To 200 mL volumes of filtrate, 200 mg of ethylenediaminetetraacetic acid was added. Then, solid-phase extraction (SPE) (Oasis HLB cartridges, 3 mL, 400 mg, Waters Corp., Milford, MA, USA) using a Phenomenex vacuum system (Torrance, CA, USA) was performed. The elutions were made with pure methanol (3 × 2 mL). The eluents were evaporated to dryness under a stream of nitrogen (99.999% purity, Multax, Poland) at 40 °C and reconstituted in a methanol-water mixture (10:90, *v*/*v*) (1 mL).

Sludge samples were centrifuged at 5000 rpm for 5 min and the supernatant was discarded. Five g of WWTP2-RS or 10 g of the other samples were placed into 50 mL polypropylene tubes, which were then mixed with 9 mL of 30 mMpotassium phosphate monobasic solution and 1 mL of methanol, and extracted for 20 min using 10 mL of acetonitrile with 1% formic acid and modified QUECHERS salts (4 g MgSO_4_, 1 g NaCl, 1 g Na_3_Citrate, and 0.5 g Na_2_Citrate•H_2_O). Next, the samples were centrifuged for 5 min at 5000 *g*. The samples were then cleaned by incubating 9 mL of extract with 500 mg octadecyl sorbent and 750 mg of MgSO_4_. They were then vortexed for 5 min at 1200 rpm and centrifuged for 5 min at 5000 rpm. Eight mL of the extract (organic layer) under a nitrogen stream at 40 °C and reconstituted in 0.5 mL of a mixture of methanol-water (10:90) was evaporated. The isotopically labeled compounds were used as an internal standards mixture to each sample before extraction (50 µL).

#### 3.2.3. Antimicrobial Detection by LC-MS/MS Analysis

Antimicrobial concentrations were analyzed by high-performance liquid chromatography coupled to mass spectrometry with a Hybrid Triple Quadrupole/Linear Ion trap mass spectrometer (QTRAP®4000, AB SCIEX, Framingham, MA, USA). LC analysis was performed using an Agilent 1260 Infinity (Agilent Technologies, Santa Clara, CA, USA) equipped with a degasser, thermostated autosampler, and binary pump, and connected in series to an AB Sciex 4000 QTRAP mass spectrometer equipped with a Turbo Ion Spray source that was operated in both positive mode and negative mode. The curtain gas, ion source gas 1, ion source gas 2, and collision gas (all high purity nitrogen) were set at 35 psi, 60 psi, 40 psi, and “medium” instrument units, respectively, and the ion spray voltage and source temperature were set at 5000 V and 600 °C, respectively. Chromatographic separation was achieved with a Kinetex RP-18 column (100 mm × 4.6 mm, 2.6 µm) supplied by Phenomenex (Torrance, CA, USA). The column was maintained at 40 °C and the flow rate was 0.5 mL/min. The mobile phase consisted of HPLC-grade water with 0.2% formic acid as eluent A and acetonitrile with 0.2% formic acid as eluent B. The gradient (%B) was as follows: 0 min. 10%, 1 min. 10%, 25 min. 90%, and 35 min. 90%. The injection volume was 10 µL. The target compounds were analyzed in multiple reaction monitoring (MRM) mode in positive ionization mode (ESI +), monitoring two transitions between the precursor ion and the most abundant fragment ions for each compound. Internal standards were attributed to analyzed compounds based on similarities between chemical structures of surrogate and analyzed compound (according to the Tanimoto similarity index). The LC-MS method was validated using three quality control levels (low, medium, and high) prepared on effluents. The interday precision higher than 15% was observed for cefadroxil (up to 22%), norfloxacin (up to 22%), lomefloxacin (up to 24%), and azithromycin (up to 35%) in case of wastewater and for cefadroxil (up to 23%), azithromycin (up to 26%), roxithromycin (up to 34%), and clarithromycin (up to 38%) in case of sediments. Significant matrix effect (lower than 85% or higher than 115%) was observed for 12 compounds in the case of wastewater and 21 in the case of sediments. Additionally to internal standard addition to control the matrix effect, each sample was tested without and after fortification with antimicrobials.

The following blanks were used: the HPLC blank (10% methanol) and the method blank (Milli-Q water, calcinated sand) to evaluate the contamination resulting from the complete preparation and analytical procedure. The positive control (water or sand fortified with pharmaceuticals) was also applied. Then, the obtained results were adjusted with recovery and matrix effect. The method detection limit (MDL) and method quantitation limit (MQL) for the entire method (including extraction) were determined as the amount of analyte in matrix spiked with signal-to-noise ratios (S/N) of 3:1 and 10:1, respectively. For the pharmaceuticals already present in samples, MDL and MQL were estimated by determining the S/N of the minimum measured concentrations and extrapolating to S/N values of 3 and 10, respectively.

### 3.3. Calculations

The following parameters were calculated:

Antimicrobial removal (RE) calculated using Equations (1) and (2) according to Douziech et al. [59]:

RE [%] = (1 − exp (RR)) × 100%(1)

RR = ln (C_EWW_/C_IWW_) (2)

where RE is the removal of antimicrobials during treatment [%], RR (response ratio (effect size)) is measured per WWTP and antimicrobial, C_EWW_ is the mean of the effluent wastewater concentration of the antimicrobial (*n* = 3), and C_IWW_ is the mean of the influent wastewater concentration of the antimicrobial (*n* = 3).

PNEC (predicted no effect concentration) for resistance selection in wastewater was calculated according to Bengtsson-Palme and Larsson [28], whereas PNEC for eukaryotic species was calculated as described by Page et al. [27]. Depending on the number of long-term toxicity tests performed, i.e., no test, the test on one, two, or three trophic levels, the lowest obtained NOEC (no observable effect level) or EC_50_ (half maximal effective concentration) was divided by 1000, 100, 50, or 10 [60]. PNEC for cyanobacteria was obtained by dividing the NOEC or EC_50_ by 10. All PNEC values were presented in Table A4. 

PNEC for soil (PNEC_soil_) and sludge (PNEC_sludge_) was obtained by multiplying PNEC with K_d_ (solid/liquid partition coefficient, Table A2). K_d_ was calculated as the ratio of the concentration of an antimicrobial on a solid phase (sludge) divided by the equilibrium concentration in the contacting liquid phase (wastewater). PNEC values for resistance selection can be found in Table A5.

Risk quotients (RQs) were calculated using Equation (3):

RQ_wastewater_ = MEC_wastewater_/PNEC (3)

RQ_sludge_ = MEC_sludge_/PNEC_sludge_ (4)

RQ_wastewater_ = PEC_soil_/PNEC_soil_ (5)

where PEC_soil_ is predicted environmental concentration in soil (Table A3), MEC is measured environmental concentration, and PNEC is predicted no effect concentration.

The risk ranking criterion was RQ < 0.1, minimal risk; 1 > RQ ≥ 0.1, medium risk; and RQ > 1, high risk [61].

Predicted daily load of the antimicrobial into WWTP (PLoad) from actual consumption data of the product using Equation (6) is:

PLoad = TOTAL·WWTP/(INHAB· 30)(6)

where PLoad is the predicted daily load of the antimicrobial into WWTP (mg/d), TOTAL is the total monthly consumption of the pharmaceutical in a country (mg), WWTP is an equivalent number of inhabitants in a WWTP, and INHAB is the number of inhabitants in a country (38,000,000).

Average daily load of the antimicrobial into a WWTP from the measured data using Equation (7) is:

Load_W+S_ = Load_W_ + Load_S_ = C_W_·Flow + C_S_·PS (7)

where Load_W+S_ is the average total daily load of antimicrobial into WWTP (mg/d), Load_W_ is the load of a compound in a water phase of an influent, Load_S_ is the load of a compound in a primary sludge, C_W_ is the concentration of antimicrobial in the WWTP influent (water phase) (mg/m^3^), Flow is the average daily flow rate in the WWTP (m^3^/d), C_S_ is the concentration of antimicrobial in the WWTP primary sludge (mg/m^3^), and PS is the average daily volume of the WWTP primary sludge (m^3^/d).

### 3.4. Statistical Analysis

The statistical analysis of the results was performed with the STATISTICA version 13.1 for Windows (TIBCO Software Inc., Palo Alto, CA, USA) and Metaboanalyst 4.0. Student’s t-test was used for comparison of samples. Principal components analysis (PCA) was used to determine differences between wastewater and sewage sludge.

## 4. Conclusions

In this study, 70% of the examined antimicrobials in both wastewater and sewage sludge collected from two Polish WWTPs were detected. To the best of our knowledge, this is the first report on antimicrobial occurrence in Polish WWTPs. The removal efficiency, antimicrobial resistance risk, and ecological threat (RQs) were examined according to the obtained data. The WWTPs removed ~50% of selected antimicrobials with good efficiency, above 50%. The level of antimicrobials in both untreated and treated wastewater, river as well as sewage sludge, poses a risk of resistance selection as shown by RQ calculations. Moreover, influents and river waters posed high and medium risk, particularly for cyanobacteria and eukaryotes due to the presence of ciprofloxacin, macrolides, and sulfamethoxazole. Following the obtained results, the watch list of substances for Union-wide monitoring in the field of water policy (already includes macrolides and ciprofloxacin) should be extended with sulfamethoxazole. Our study also indicates the need for evaluation of antimicrobials’ concentrations not only in treated wastewater, but also in sewage sludge because of its usage in the fertilization process, which is environmentally sustainable options for re-use of the WWTP by-products. Several antimicrobials tested were present at levels that have been suggested to promote resistance development in sludge-amended soils (predicted concentrations).

The most important observation made is a possible pressure for the development of antimicrobial resistance in the WWTPs. WWTPs can be considered as potential hot spots for the dissemination of antimicrobial resistance. Leakage of antimicrobials can select for increased resistance among environmental bacteria and influence the virulence of antimicrobial-sensitive bacterial infections directly by reducing the infective dose and transmission [27,29]. Therefore, additional studies on the characterization of wastewater treatment plants’ microbial communities and the profiles of antimicrobial-resistant genes are necessary. Our study also highlights the lack of sufficient data to evaluate or predict the risk of resistance development and environmental threat. In fact, data on risk assessment of wastewater and sludge in other European countries is also scarce.

## Figures and Tables

**Table 1:** Mean and standard deviation (*n* = 3) of the target antimicrobial concentrations (ng/L) in influent, effluent, and receiving water of two wastewater treatment plants (WWTPs) located in Poland at Silesian (WWTP1) and Warmian-Masurian Voivodship (WWTP2).

	WWTP1 Influent		WWTP1 Effluent		WWTP1 Leachate		River1 Upstream		River1 Downstream		WWTP2 Influent		WWTP2 Effluent		River2 Upstream		River2 Downstream		MDL ^1^	MQL ^1^
	Mean	SD	Mean	SD	Mean	SD	Mean	SD	Mean	SD	Mean	SD	Mean	SD	Mean	SD	Mean	SD		
AZM	87	71	230	110	320	290	25	15	441	249	360	450	650	680	5.2	2.7	36	21	10	33
CIP	1260	680	184	72	890	410	108	33	95	4	4300	4300	312	73	12	12	182	182	2.4	8.1
CLR	480	190	160	170	102	23	37	9	79	50	560	590	143	40	12.2	8.2	20.5	11.1	0.3	0.9
CLI	134	87	166	60	73	37	78	46	134	3	106	51	290	200	2.3	1.1	25.4	3.5	2.9	9.5
ERY	58	71	21	18	30	42	7	7	10	0	28	20	16	12	<MDL		<MDL		0.7	2.4
LCM	20	15	48	52	24.7	7.8	3	3	9	4	102	46	56	19	<MDL		4.0	1.4	1.4	4.7
MTZ	250	160	69	82	11.1	8.4	9	3	21	11	7400	9600	88	41	<MDL		9.4	3.1	2.4	7.9
NOR	240	130	31	28	210	150	95	93	<MDL		80	110	10	11	<MDL		<MDL		6.3	21
OFX	135	35	26	14	200	91	32	18	4	4	195	21	40	11	8.4	0.5	31	23	1.4	4.6
OTC	0.7	0.9	0.1	0.1	1.1	1.4	<MDL		1	0	<MDL		<MDL		<MDL		<MDL		0.1	0.2
RIF	5.2	3.3	<MDL		<MDL		<MDL		<MDL		5.3	3.4	2.9	2	<MDL		<MDL		2.9	9.6
ROX	18	19	6.6	6.9	1.6	1.6	4	4	5	2	6.2	5.3	6.2	2.1	<MDL		<MDL		0.5	1.6
SD	<MDL		3.4	4.3	2.4	2.5	<MDL		<MDL		<MDL		<MDL		<MDL		<MDL		0.6	1.9
SDM	4.1	3.2	3.4	3.5	<MDL		<MDL		<MDL		8.9	7.2	4.9	3.6	<MDL		<MDL		1.8	6.1
SXT	1300	460	630	220	480	630	644	41	451	95	3000	1900	770	280	<MDL		76.1	4.6	5.9	19
ST	94	46	21	14	136	54	7	6	29	19	180	110	36	16	<MDL		<MDL		2.4	8
TET	190	190	39	55	180	170	<MDL		0	0	210	160	61	48	<MDL		7.2	6.4	0.2	0.7
TBZ	18.4	2.8	22.3	4.5	18	11	12	7	16	0	11	2.8	25.5	3	<MDL		4.3	2.1	2.8	9.4
TMP	254	41	160	190	94	52	38	3	36	8	900	770	220	110	9.1	8.4	24.4	3.9	3	9.9
VAN	350	390	114	60	840	220	27	23	62	8	3200	3600	162	62	<MDL		10.7	3.2	15	50

CFR (270 ng/L), FLRX (3.1 ng/L), LOM (0.9 ng/L), NAL (6.1 ng/L), PEF (12 ng/L), SDD (3.7 ng/L) were not detected. Their MDLs (method detection limits) are provided in parentheses. ^1^ Presented MDLs and method quantitation limits (MQLs) were calculated for influents. The values for effluents are about two times lower and for surface water about four times lower.

**Table 2:** Mean and standard deviation (*n* = 3) of target antimicrobial concentration (ng/g dry weight) in sewage sludge samples in two wastewater treatment plants (WWTPs) located in Poland at Silesian (WWTP1) and Warmian-Masurian Voivodship (WWTP2).

	WWTP1-PS		WWTP1-ES		WWTP2-RS		WWTP2-FS		WWTP1-RS ^1^		MDL	MQL
	Mean	SD	Mean	SD	Mean	SD	Mean	SD	Mean	SD		
AZM	1260	690	370	280	82	75	1040	940	100	110	23	79
CIP	12500	6900	6200	2000	6100	5500	28000	22000	7800	4400	490	1670
CLR	289	52	18	14	18	23	150	180	3.0	0.8	2.2	7.4
CLI	40	25	58	35	29	29	47	30	9.1	5.6	1.3	4.5
LCM	4.3	3.2	1.5	2.0	2.5	2.6	19	24	0.13	0.05	0.2	0.6
NOR	5300	3200	3600	2500	1600	1400	3400	2300	<MDL		370	1270
OFX	810	890	640	510	300	190	480	120	36	21	12	41
OTC	590	410	320	230	50	35	190	200	4.0	6.5	0.2	0.7
PEF	250	150	190	110	140	120	256	81	<MDL		29	100
ROX	11.4	7.8	<MDL		<MDL		<MDL		0.22	0.26	12	40
SD	<MDL		<MDL		1.1	0.8	7.7	11	0.14	0.18	1.1	3.7
SDM	12	14	13	14	7.5	11	<MDL		0.29	-	0.6	2.0
SXT	<MDL		81	54	<MDL		<MDL		5.9	9.4	41	140
TET	82	50	40	13	58	29	201	95	25	29	0.7	2.5
TBZ	19.5	5.2	15.6	5.8	43	43	49	22	8.3	6.2	0.6	2.2
TMP	170	110	5.7	4.4	<MDL		<MDL		2.6	1.7	0.5	1.5
VAN	363	55	181	91	36	30	<MDL		370	130	32	110

CFR (350 ng/g), FLRX (12 ng/g), LOM (7.9 ng/g), NAL (3.3 ng/g), MTZ (25 ng/g), RIF (150 ng/g), ST (30 ng/g), SDD (39 ng/g) were not detected. Their MDLs (method detection limits) are provided in parentheses; MQL, method quantitation limit; WWTP-PS, primary sludge; WWTP-ES, excessive sludge; WWTP-FS, fermented sludge, WWTP-RS residual sludge; ^1^calculated based to the lowest K_d_ observed and concentration of antimicrobials in leachate.

## Appendix A

**Table A1:** The comparison of the predicted (PLoad) and measured load of antimicrobial into WWTP1.

	Sale in 2018 (kg/month)	PLoad (g/d) ^1^			Load_W_ (g/d) ^2^			Load_S_ (g/d) ^3^			Load_W_/PLoad (%)			Load_S_/PLoad (%)			Load_W+S_ ^4^/PLoad(%)		
	range	S^5^	A	W	S	A	W	S	A	W	S	A	W	S	A	W	S	A	W
AZM^6^	98–352	25.3	26.3	47.2	0.3	4.8	1.9	0.2	2.9	5.4	1	13	4	1	8	11	2	21	15
CIP	601–821	106.5	128.9	112.8	58.7	15.6	27.1	54.2	26.1	60.1	55	12	24	51	20	53	106	32	77
CLR	288–1509	68.9	121.1	145.5	7.9	11.2	19.4	0.04	0.27	0.09	12	9	13	0	0	0	12	9	13
CLI	608–737	109.9	116.4	106.8	1.6	2.4	6.8	0.3	0.2	0.8	1	2	6	0	0	1	1	2	7
NOR	179–214	30.9	35.5	29.9	5.1	11.2	3.0	10.3	18.0	52.7	16	31	10	33	51	176	49	82	186
OFX	609–829	107.3	131.0	113.7	2.6	4.9	3.5	10.2	1.5	2.8	2	4	3	10	1	3	12	5	6
RIF	40–48	7.08	7.19	6.63	0.26	0.08	0.08				4	1	1	0	0	0	4	1	1
ROX	6–28	1.44	2.33	2.69	0.14	0.14	1.19				10	6	44	0	0	0	10	6	44
SXT	606–1358	133.3	196.0	194.1	18.5	44.9	44.0	0.4	0.3	1.2	14	23	23	0	0	1	14	23	24
TMP	122–272	26.7	39.7	38.9	8.1	5.5	6.8	0.02	0.02	0.09	30	14	18	0	0	0	30	14	18

^1^ PLoad, predicted load of a compound calculated on the basis of sales data; ^2^ Load_W_, load of a compound calculated on the basis of measured concentration in a water phase of an influent; ^3^ Load_S_, load of a compound calculated on the basis of measured concentration in a primary sludge; ^4^ Load_W+S_, total load of a compound (water phase and a primary sludge); ^5^ S summer; A, autumn; W, winter sampling. ^6^ The pharmaceuticals used only in hospitals and frequently used in veterinary medicine were not presented

**Table A2:** Mean values of sorption coefficients (log K_d_) calculated for sludge and the percent of the total mass of the compound sorbed in sludge to its total daily mass load to WWTP.

Antimicrobial	% of Total Mass Load Sorbed to Sludge	log K_d_ Sludge
AZM	73%	7.1 ± 4.3
CIP	55%	7.3 ± 4.2
CLR	18%	5.7 ± 2.5
CLI	9%	5.7 ± 2.6
LCM	7%	5.2 ± 1.9
OFX	43%	7.7 ± 4.8
OTC	62%	9.8 ± 6.9
SDM	32%	6.8 ± 3.4
SXT	2%	5.1 ± 1.7
TET	13%	8.3 ± 5.5
TBZ	23%	5.9 ± 2.6
TMP	19%	4.8 ± 1.6
VAN	24%	6.3 ± 3.2

**Table A3:** Predicted antimicrobial concentration in sludge-amended soil (single sludge application).

Antimicrobial (ng/g)	WWTP1-PS		WWTP1-ES		WWTP2-RS		WWTP2-FS		WWTP1-RS ^1^	
	Mean	SD	Mean	SD	Mean	SD	Mean	SD	Mean	SD
AZM	1.9	1.3	0.55	0.50	0.17	0.14	2.3	1.6	0.14	0.13
CIP	18	13	9.1	3.5	8.9	9.8	41	40	11.4	5.2
CLR	0.42	0.09	0.03	0.02	0.03	0.04	0.33	0.37	<0.01	-
CLI	0.06	0.04	0.08	0.06	0.04	0.05	0.07	0.05	0.01	0.01
LCM	0.01	0.01	0.01	-	0.01	0.01	0.03	0.04	<0.01	-
NOR	7.8	5.8	5.2	4.4	2.4	2.4	5.0	4.1	-	-
OFX	1.2	1.6	0.94	0.91	0.44	0.33	0.70	0.21	0.05	0.02
OTC	0.86	0.74	0.48	0.41	0.07	0.06	0.41	0.37	0.01	0.01
PEF	0.36	0.25	0.28	0.20	0.28	0.22	0.38	0.15	-	-
ROX	0.03	-	<0.01	-	<0.01	-	<0.01	-	<0.01	-
SD	<0.01		<0.01		<0.01	-	0.03	-	<0.01	-
SDM	0.03	0.03	0.03	0.03	0.03	-	<0.01	-	<0.01	-
SXT	<0.03	-	0.12	0.10	<0.03	-	<0.03	-	0.01	0.01
TET	0.12	0.09	0.06	0.02	0.08	0.05	0.29	0.17	0.04	0.03
TBZ	0.03	0.01	0.02	0.01	0.09	0.08	0.07	0.04	0.01	0.01
TMP	0.25	0.19	0.01	0.01	<0.01	-	<0.01	-	<0.01	-
VAN	0.53	0.10	0.27	0.16	0.11	-	<0.01	-	0.55	0.15

^1^ WWTP-PS, sludge after mechanical treatment; WWTP-ES, sludge after gravity treatment; WWTP-FS, sludge after an open fermentation pool; WWTP-RS, sludge for management.

**Table A4:** NOEC/EC_50_ (μg/L) and PNEC (ng/L) used for the risk assessment; nd, no data available. Abbreviations in parentheses indicate the most sensitive taxa: MA, microalgae; MP, macrophytes; IN, invertebrates. The data based on the review of Le Page [27].

Compound	Cyanobacteria NOEC/EC_50_	Cyanobacteria PNEC	Eukaryote NOEC/EC_50_	Eukaryote PNEC
AZM	0.19	19	1.8	180(MA)
CIP	5.65	565	10	1000(MP)
CLR	0.84	84	2	40(MA)
ERY	2	200	10.3	206(MA)
LCM	18	1800	548	10976(MA)
MTZ	0	nd	250,000	5000000(IN)
NOR	1.6	160	300	3000(MP)
OFX	5	500	31.2	624(MA)
OTC	3.1	310	183	3660(MA)
ROX	nd	nd	10	100(MA)
SD	3900	390,000	135	2700(MA)
SDM	7800	780,000	100	5290(MA)
SXT	5.9	590	10	1000(MP,IN)
TET	90	9000	300	6000(MP)
TMP	1385	135,800	1000	20000(MP)

**Table A5:** Predicted no effect concentration values for resistance selection of microbial community in wastewater, sludge, and soil.

Antimicrobial	PNEC Wastewater (µg/L) ^1^	PNEC Sludge (µg/kg) ^2^	PNEC Soil (µg/kg) ^3^
AZT	0.25	1200	47
CIP	0.064	1200	27,000
CLR	0.25	140	66
CLI	1	280	-
ER	2	-	260
LIN	2	340	-
METR	0.125	-	0.07
NFL	0.5	-	300
OFL	0.5	4200	730
OTET	0.5	4,200,000	210
RIF	0.5	-	-
ROX	1	4300	50
STH	16	2000	9.6
TET	1	3000	1100
TRI	0.5	100	3.7
VAN	8	17,000	2.4

^1^ According to Bengtsson-Palme and Larsson [28]; ^2^ calculated based on K_d_ obtained for sludge; ^3^ calculated according to [26,40,42,62].
